# STR-realigner: a realignment method for short tandem repeat regions

**DOI:** 10.1186/s12864-016-3294-x

**Published:** 2016-12-03

**Authors:** Kaname Kojima, Yosuke Kawai, Kazuharu Misawa, Takahiro Mimori, Masao Nagasaki

**Affiliations:** Tohoku Medical Megabank Organization, Tohoku University, 2-1, Seiryo-machi, Aoba-ku, Sendai, 980-8573 Japan

**Keywords:** High-throughput sequencing, Short tandem repeat, Alignment

## Abstract

**Background:**

In the estimation of repeat numbers in a short tandem repeat (STR) region from high-throughput sequencing data, two types of strategies are mainly taken: a strategy based on counting repeat patterns included in sequence reads spanning the region and a strategy based on estimating the difference between the actual insert size and the insert size inferred from paired-end reads. The quality of sequence alignment is crucial, especially in the former approaches although usual alignment methods have difficulty in STR regions due to insertions and deletions caused by the variations of repeat numbers.

**Results:**

We proposed a new dynamic programming based realignment method named STR-realigner that considers repeat patterns in STR regions as prior knowledge. By allowing the size change of repeat patterns with low penalty in STR regions, accurate realignment is expected. For the performance evaluation, publicly available STR variant calling tools were applied to three types of aligned reads: synthetically generated sequencing reads aligned with BWA-MEM, those realigned with STR-realigner, those realigned with ReviSTER, and those realigned with GATK IndelRealigner. From the comparison of root mean squared errors between estimated and true STR region size, the results for the dataset realigned with STR-realigner are better than those for other cases. For real data analysis, we used a real sequencing dataset from Illumina HiSeq 2000 for a parent-offspring trio. RepeatSeq and lobSTR were applied to the sequence reads for these individuals aligned with BWA-MEM, those realigned with STR-realigner, ReviSTER, and GATK IndelRealigner. STR-realigner shows the best performance in terms of consistency of the size of estimated STR regions in Mendelian inheritance. Root mean squared error values were also calculated from the comparison of these estimated results with STR region sizes obtained from high coverage PacBio sequencing data, and the results from the realigned sequencing data with STR-realigner showed the least (the best) root mean squared error value.

**Conclusions:**

The effectiveness of the proposed realignment method for STR regions was verified from the comparison with an existing method on both simulation datasets and real whole genome sequencing dataset.

## Background

From the development of high-throughput sequencing (HTS) technologies, the detailed variant detection is enabled for each individual with whole genome sequencing analysis. For single nucleotide variants (SNVs), various types of variant calling methods have been proposed [1–4] for HTS data, and the accurate SNV detection is archived for more than a thousand of individuals in genome-wide scale [5, 6]. However, unlike SNVs, there still exists difficulty in the accurate detection of structural variations such as genome insertion, genome deletion, short tandem repeat (STR) number polymorphisms, and copy number variations, especially from data with low read coverage [7].

For repeat number polymorphisms, several studies thus far reported associations with various disease phenotypes such as CAG repeats in the Huntingtin gene with Huntington’s disease [8] and CAG repeats in the androgen receptor gene with spinal and bulbar muscular atrophy [9]. From HTS data, several approaches such as lobSTR [10], RepeatSeq [11], STRViper [12], and coalescentSTR [13] have been proposed for estimating repeat numbers in STR regions. In lobSTR and RepeatSeq, repeat patterns included in sequence reads spanning the STR regions are considered for the estimation of repeat numbers. On the other hand, STRViper and coalescentSTR estimate repeat numbers by considering difference between the actual insert size and the insert size inferred from paired-end reads aligned to the flanking regions of the target repeat. The alignment quality of sequence reads is important for accurate repeat number estimation, especially in the former approaches although usual alignment methods have difficulty in STR regions due to insertions and deletions caused by the frequent change of repeat numbers.

We propose a new dynamic programming based realignment method named STR-realigner where repeat patterns in STR regions are given as prior knowledge, and repeat patterns are used multiple times in the realignment process. Although a similar algorithm is adopted in a tool for detecting STR regions in PacBio reads based on 3-stage modified Smith-Waterman [14], consecutive STR regions can be handled in the proposed algorithm unlike the tool. In addition, clipping fragments, which are an essential feature for the realignment, are also considered in the proposed algorithm. By allowing insertions and deletions of repeat patterns in STR regions with repeatedly use of repeat units, accurate realignment of sequence reads is expected.

In a simulation study with synthetically generated HTS data for artificial diploid genomes sequence based on phased genotypes of a sample in the dataset of 1000 Genomes Project [5], we showed the effectiveness of our model by evaluating root mean squared errors between true and estimated repeat numbers with RepeatSeq or allelotype, an STR calling software in the lobSTR package, from realignment results. For real data analysis, we applied STR-realigner, ReviSTER [15], and GATK IndelRealigner to HTS data from Illumina HiSeq 2000 for a HapMap CEU parent-offspring trio and show the effectiveness of STR-realigner based on consistency in Mendelian inheritance in the estimated repeat numbers in the parent-offspring trio. Root mean squared error values were also calculated from the comparison with the gold standard STR region size obtained from high coverage PacBio sequencing data for one of samples in the parent-offspring trio, and the results from the realigned sequencing data with STR-realigner showed the least (the best) root mean squared error value.

## Method

### Realignment algorithm considering repeat sequence as prior knowledge

**Fig. 1:**
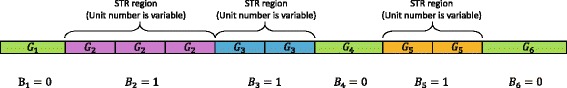
An example of notations in STR-realigner. Subsequence with *B*_*j*_=1 can be used repeatedly in the realignment process

We propose a dynamic programming based algorithm named STR-realigner that realigns query read *R* to a genome sequence, taking into account the multiple use of repeat patterns for prespecified STR regions. We consider a genome sequence comprised of series of *m* subsequences *G*_1_,…,*G*_*m*_. Let *B*_*j*_ be a binary variable that takes one if *G*_*j*_ can be used repeatedly and zero otherwise, i.e., subsequence *G*_*j*_ with *B*_*j*_=1 is for a repeat pattern in one of prespecified STR regions. Figure 1 shows an example of a genome sequence comprised of subsequences *G*_1_,…,*G*_6_, where *G*_2_, *G*_3_, and *G*_5_ are repeat patterns of prespecified STR regions and are repeatedly used in the proposed realignment algorithm. In the description of the proposed algorithm, |*R*| and |*G*_*j*_| denote the size of *R* and *G*_*j*_, and *R*[ *k*] and *G*_*j*_[ *k*] denote bases at the *k*th position of *R* and *G*_*j*_, respectively.

Since infinitely long deletions can be considered by using the same subsequence with *B*_*j*_=1 repeatedly, we limit the size of deletions to less than |*G*_*j*_| for subsequences with *B*_*j*_=1. We consider the following six types of states for the alignment of the *i*th position in query read *R* to the *k*th position of subsequence *G*_*j*_.

1. *s*_*M*_(*i,j,k*): a state representing match or mismatch between bases at the *i*th position of query read *R* and the *k*th position of subsequence *G*_*j*_.
2. *s*_*I*_(*i,j,k*): a state representing insertion at the *i*th position of query read *R* right after the *k*th position of subsequence *G*_*j*_.
3. *s*_*D*_(*i,j,k*): a state representing deletion of the *k*th position of subsequence *G*_*j*_ right after the *i*th position of query read *R*.
4. *s*_*D*_(*i,j,k,l*): a state representing deletion from the *k*−*l*+1 to *k*th positions of subsequence *G*_*j*_ right after the *i*th position of query read *R*. This state is considered only for subsequences with *B*_*j*_=1 in order to avoid deletions longer than |*G*_*j*_| by limiting the range of *l* from 2 to |*G*_*j*_|−1. For *l*=1, *s*_*D*_(*i,j,k*) is used, and consecutive deletions in the same subsequence are not considered for *s*_*D*_(*i,j,k*) with *B*_*j*_=1. If *l* is longer than *k*, the deletion starts from the |*G*_*j*_|−*l*−*k*+1st position on the subsequence and the deletion part rotates from tail to head of the subsequence.
5. *s*_*L*_(*i*): a state representing left clipping that ends at the *i*th position of query read *R*.
6. *s*_*R*_(*i*): a state representing right clipping that starts at the *i*th position of query read *R*.

The following penalties are considered in the proposed realignment algorithm.

- *p*_*m,j*_: penalty for match of bases between query read *R* and subsequence *G*_*j*_. Usually, the penalty is set to a minus value, i.e., the penalty is used for rewarding.
- *p*_*mis,j*_: penalty for mismatch of bases between query read *R* and subsequence *G*_*j*_.
- *p*_*io,j*_ and *p*_*ie,j*_: penalties for open and extension of insertion on subsequence *G*_*j*_, respectively.
- *p*_*do,j*_ and *p*_*de,j*_: penalties for open and extension of deletion on subsequence *G*_*j*_, respectively.
- *p*_*c*_: penalty for clipping.

In the proposed dynamic programming algorithm, penalty and traceback information for state *s* are stored in functions *P*(*s*) and *T*(*s*), respectively. In the first step of the dynamic programming, penalty and traceback information of states for the first position in query read *R* are initialized in the following algorithm.



The best penalties for the alignment up to the *i*th position of query read *R* for each state is updated by using the best penalties of states for the *i*−1st position of query read *R* in Algorithm 2, where traceback information is also updated. Algorithm 3 given below updates penalty and traceback information for states representing match or mismatch. Algorithm 4 given below is used for obtaining states that are in preceding subsequences and can be traced from *s*_*M*_(*i,j*,1). Algorithm 5 given below updates penalty and traceback information for states representing insertion. Algorithm 6 given below updates penalty and traceback information for states representing deletion.











For subsequence *G*_*j*_ with *B*_*j*_=1, consecutive deletions in the same subsequence are handled with *s*_*D*_(*i,j,k,l*), and hence *s*_*D*_(*i*−1,*j,k*−1) is not considered at step 6 of Algorithm 6 for traceback. Procedures for updating penalty and traceback information for states representing consecutive deletions for subsequence *G*_*j*_ with *B*_*j*_=1 is given as Algorithm 7. Algorithm 8 given below updates penalty and traceback information for *s*_*R*_(*i*). Finally, an algorithm for traceback is given as Algorithm 9. By following states from head to tail in *Q* obtained with the above algorithm, the realignment result with the best penalty is obtained.







**Fig. 2:**
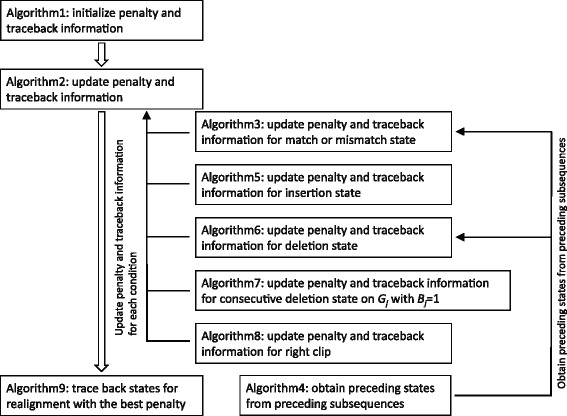
A flowchart of algorithms considered in STR-realigner. After initialization of penalty and traceback information for first query position with Algorithm 1, penalty and traceback information are updated for other query positions with Algorihtm 2 in a dynamic programming manner. Then, a realignment with the best penalty is obtained from traceback information with Algorithm 9

Figure 2 summarize a relationship of the above nine algorithms considered in STR-realigner as a flowchart. After initialization of penalty and traceback information for first query position with Algorithm 1, penalty and traceback information are updated for other query positions with Algorihtm 2 in a dynamic programming manner. Then, a realignment with the best penalty is obtained from traceback information with Algorithm 9.

### Time and space complexities of STR-realigner

#### Time complexity analysis

For each position *i* in query read *R*, updating penalty and traceback information takes *O*(1) time for *s*_*M*_(*i,j,k*), *s*_*I*_(*i,j,k*), and *s*_*D*_(*i,j,k*) for *k*>1 and subsequence *G*_*j*_ with *B*_*j*_=0. For *k*>1 and subsequence *G*_*j*_ with *B*_*j*_=1, updating information for *s*_*M*_(*i,j,k*) and *s*_*I*_(*i,j,k*) requires *O*(|*G*_*j*_|) time while updating information for *s*_*D*_(*i,j,k*) and *s*_*D*_(*i,j,k,l*) requires *O*(1) time. For *k*=1, states for tail positions of preceding subsequences are additionally considered until reaching to subsequence *G*_*j*_ with *B*_*j*_=0 or *j*=1 as in Algorithm 4. This process additionally requires $O\left (\sum _{x={j'}}^{j} |G_{x}|\right)$ time for *s*_*M*_(*i,j*,1), where *j* ^′^ is one or the index for the first subsequence *Gj*′ with *Bj*′=0 reached from *G*_*j*_. However, since the best state and its corresponding penalty before *G*_*j*−1_ are already considered for updating information for *s*_*M*_(*i,j*−1,1), by using this information, we need to newly consider only states in subsequence *G*_*j*−1_, and hence the additionally required time complexity is reduced to *O*(|*G*_*j*−1_|). Thus, with the modification of the algorithm according to the above argument, updating information for states *s*_*M*_(*i*,1,1),…,*s*_*M*_(*i,m*,1) requires $O\left (\sum _{j} |G_{j}|\right)$ time in total. Since the same optimization can be applied to updating information for states representing insertion, updating information for states with *k*=1 requires $O\left (\sum _{j} |G_{j}|\right)$ time in total as well. In addition, for *s*_*L*_(*i*) and *s*_*R*_(*i*), *O*(1) time and $O\left (\sum _{j} |G_{j}|\right)$ time are required, respectively. Thus, updating penalties and traceback information for all the states requires $O\left (\sum _{j} |G_{j}| + \sum _{j \in \{j'| B_{j'} = 1\}} |G_{j}|^{2}\right)$ time for each position in query read *R*, and hence the time complexity of the proposed algorithm is $O\left (|R| \cdot \left (\sum _{j} |G_{j}| + \sum _{j \in \{j'| B_{j'} = 1\}} |G_{j}|^{2}\right)\right)$ time.

#### Space complexity analysis

The order of the number of states for each position in query read *R* is $O\left (\sum _{j} |G_{j}|\right)$ for *s*_*M*_(*i,j,k*), *s*_*I*_(*i,j,k*), and *s*_*D*_(*i,j,k*). For *s*_*D*_(*i,j,k,l*), the order is $O\left (\sum _{j \in \{j'| B_{j'} = 1\}} |G_{j}|^{2}\right)$, and for *s*_*L*_(*i*) and *s*_*R*_(*i*), the order is *O*(1). Thus, storing values from functions *P* and *T* requires $O\left (|R| \cdot \left (\sum _{j} |G_{j}| + \sum _{j \in \{j'| B_{j'} = 1\}} |G_{j}|^{2}\right)\right)$ space. However, *P*(*s*_*D*_(*i,j,k,l*)) can be obtained by calculating *P*(*s*_*D*_(*i,j,k*))+(*l*−1)·*p*_*de,j*_, and *T*(*s*_*D*_(*i,j,k,l*)) is given by *s*_*D*_(*i,j,k,l*−1) for *l*>2 and *s*_*D*_(*i,j,k*) for *l*=2. Thus, the order of the space required for functions *P* and *T* can be reduced to $O\left (|R| \cdot \left (\sum _{j} |G_{j}|\right)\right)$ by calculating functions *P* and *T* for *s*_*D*_(*i,j,k,l*) with *O*(1) time when their values are required. The space required for updating for each state is less than the order of the number of states and is negligible, compared to spaces required for *P* and *T*. Thus, with the above modification, the proposed algorithm requires $O\left (|R| \cdot \left (\sum _{j} |G_{j}|\right)\right)$ space.

### Practical implementation

Irregular repeat patterns are often contaminated in the provided STR regions detected by some Bioinformatics tools [16, 17], and those irregular repeat patterns worsen the quality of the alignment of the proposed algorithm due to the difference of the actual sequence and the assumed repeat pattern. In order to address this issue, we extract maximal regions containing repeat patterns consecutively with some pre-specified error rate from the target STR region. The extracted region is used for a new target STR region for STR-realigner.

In order to use the realignment result from the proposed algorithm for resequenced data, parts of the query read aligned to *G*_*j*_ with *B*_*j*_=1 are again realigned to the corresponding STR region of the reference genome. However, the quality of the alignment is also worsened due to irregular patterns in the STR region. Thus, we consider a subsequence for a repeat pattern right after the target STR region and set lower deletion penalty to the target STR region. For penalty, the following setting were used in our study: *p*_*m,i*_=−1, *p*_*mis,i*_=4, *p*_*io,i*_=6, *p*_*ie,i*_=1, *p*_*do,i*_=6, *p*_*de,i*_=1, and *p*_*c*_=5. These parameter values are the same as the default values in BWA-MEM. For subsequences corresponding to target STR regions for lower deletion penalty, *p*_*do,i*_ is set to 4.

**Fig. 3:**
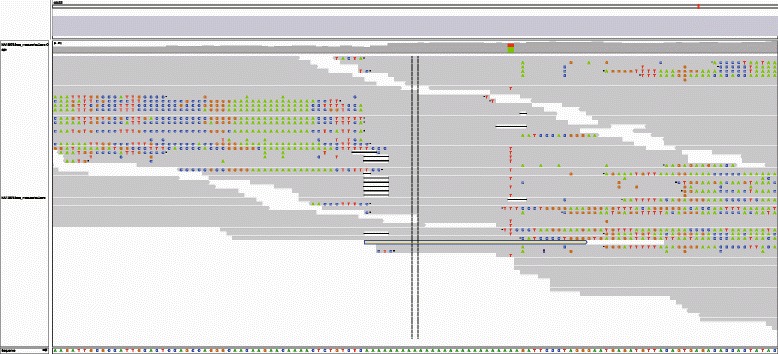
An alignment result around a homopolymer region. Most of the reads spanning the region contain soft clipping parts due to drastic sequencing errors after the homopolymer region

In Illumina reads, bases at positions after homopolymer regions are highly erroneous because the same phasing is accumulated in synthesis during the Illumina sequencing process in homopolymer regions. Figure 3 shows an example of erroneous bases around a homopolymer region where a lot of clippings occur around a long homopolymer comprised of A bases in GRCh37 due to sequencing errors. Since sequence reads with such highly erroneous bases worsen the quality of realignment with STR-realigner, we additionally implemented an option that skips the realignment with STR-realigner for homopolymer regions with some specified size such as 15.

Each mapping tool has its specific characteristics in the aligned reads. For example, a deletion exists in the start position of an STR region in the reads aligned with some mapping tool while a deletion exists in the end position of the STR region in the alignment result of another mapping tool for the same sequence reads. The performance of variant calling is worsened if such characteristics are mixed in the alignment results. Thus, all the reads aligned to a target STR region are realigned with STR-realigner in the default condition.

## Results and discussion

### Simulation analysis

From a list of STR regions provided in the RepeatSeq software package, we extracted STR regions for evaluation as follows:

- STR regions not in chromosome 22 were filtered out.
- STR regions with size longer than 100 bp were filtered out.

The maximum period, the size of repeat pattern, in the list is six. Since the length of sequence reads considered in the following experiments is 100 or 101 bp and these sequence reads cannot span STR regions > 100 bp for most of the cases, STR regions > 100 bp were filtered out. We then prepared synthetically generated diploid genome sequences of chromosome 22 based on phased genotypes for a CEU individual, NA12286, in the phase3 phased reference panel by the 1000 Genomes Project [18]. In the generation of the above genome sequences, variants located in the extracted repeat regions were ignored. The number of variants in total is 54,897. By randomly sampling repeat numbers, we generated two sets of repeat numbers for the extracted repeat regions and added STR variants to the diploid genome sequences based on the sets of repeat numbers for the evaluation. Note that repeat numbers with which the size of STR region is > 100 bp were avoided in the random sampling process. From the diploid genome sequences, we generated paired-end sequence reads in FastQ format with the read length of 100 bp and the insert size normally distributed with mean of 500 bp and standard deviation of 50 bp. In the generated reads, substitution errors were added with rate of 0.1%. Base quality scores for bases in FastQ format were set to Q30, which corresponds to 0.1% error. The read coverage of the generated data is 40 ×. A BAM file for the dataset was obtained by mapping the sequence reads to the reference genome (GRCh37) with BWA-MEM (0.7.12-r1039) [19]. We applied our proposed realignment method, STR-realigner, ReviSTER (0.1.7), and GATK IndelRealigner (GATK 3.4-0) to the BAM independently and generated three types of BAM files.

For GATK IndelRealigner, USE_READS was used for --consensusDeterminationModel option. RepeatSeq (v0.8.2) was applied to the original BAM file and the three types of realigned BAM files, and sizes of variants in the target STR regions were obtained. Table 1 shows call rates of results from RepeatSeq using the original BAM file and the three types of realigned BAM files. The call rate indicates the rate of results with STR region size estimated as a non-NA value. For all the STR periods other than period of 1, call rates of results from the BAM file realigned with STR-realigner are higher than those from other BAM files.

**Table 1:** Call rate of STR calling results with RepeatSeq using the original BAM file of 40 × and those realigned with STR-realigner, ReviSTER, and GATK IndelRealigner. The best result is underlined

Period	No. of regions	STR-realigner	ReviSTER	IndelRealigner	Original BAM
1	5345	0.878	0.878	0.873	0.872
2	1160	0.799	0.794	0.785	0.784
3	517	0.834	0.807	0.799	0.803
4	1433	0.840	0.766	0.771	0.783
5	668	0.856	0.811	0.819	0.819
6	472	0.881	0.850	0.852	0.856
Total	9595	0.859	0.841	0.838	0.840

Table 2 summarizes the root mean squared errors (RMSE) between estimated and true STR region size for each BAM file for all the STR regions. In the calculation of RMSE, the size in the reference genome was assigned for the region size estimated as NA value. For all the STR periods other than period of 1, the results from the BAM file realigned with STR-realigner show the best RMSE value. The RMSE value from the results based on the BAM file realigned with GATK IndelRealigner is slightly better than that based on the original BAM file. In order to examine the performance excluding the results estimated as NA value, we summarized RMSE for STR regions where results were commonly estimated as a non-NA value on all the four types of BAM files in Table 3. Similarly to the results for all the STR regions, the results from the BAM file realigned with STR-realigner show the best RMSE value for all the STR periods other than period of 1. The RMSE value from the results based on the BAM file realigned with GATK IndelRealigner is slightly better than that based on the original BAM file. We also applied allelotype (4.0.0) [10], an STR calling software in lobSTR package, to the original BAM file and the three types of realigned BAM files. Tables 4, 5 and 6 show call rates, RMSE values averaged on all the regions, and RMSE values averaged on commonly called regions for results from allelotype, respectively. In these tables, the results for the original BAM file realigned by allelotype with --realign option are also included. The results for the BAM files realigned with STR-realigner and ReviSTER gave the highest call rate for all the STR periods other than period of 4 and the case considering all the periods. The results for STR-realigner gave the best RMSE value for STR periods of 2, 4, and 6 and the case considering all the periods for commonly called regions although the results for STR-realigner are slightly worse than those for ReviSTER in total for Table 5.

**Table 2:** Root mean squared error (RMSE) between true and estimated repeat numbers with RepeatSeq using the original BAM file of 40 × and those realigned with STR-realigner, ReviSTER, and GATK IndelRealigner for all the STR regions. The best result is underlined

Period	No. of regions	STR-realigner	ReviSTER	IndelRealigner	Original BAM
1	5345	3.726	2.700	8.273	8.461
2	1160	2.648	4.648	8.213	8.539
3	517	2.151	4.022	8.242	8.601
4	1433	3.199	5.726	9.523	9.597
5	668	3.885	6.701	10.156	10.325
6	472	2.431	5.976	10.185	10.437
Total	9595	3.421	4.162	8.705	8.900

**Table 3:** Root mean squared error (RMSE) between true and estimated repeat numbers with RepeatSeq using the original BAM file of 40 × and those realigned with STR-realigner, ReviSTER, and GATK IndelRealigner for commonly called STR regions. The best result is underlined

Period	No. of regions	STR-realigner	ReviSTER	IndelRealigner	Original BAM
1	4659	3.858	2.718	8.694	8.891
2	900	2.471	4.815	8.735	9.084
3	410	2.402	3.265	8.548	8.930
4	1084	3.152	4.428	9.688	9.796
5	536	3.600	5.855	10.219	10.422
6	399	2.248	5.593	10.498	10.793
Total	7988	3.484	3.741	9.038	9.253

**Table 4:** Call rate of STR calling results with allelotype using the original BAM file of 40 × and those realigned with STR-realigner, ReviSTER, GATK IndelRealigner, and allelotype with --realign option. The best result is underlined

Period	No. of regions	STR-realigner	ReviSTER	IndelRealigner	--realign option	Original BAM
1	5345	1.000	1.000	0.998	0.998	0.998
2	1160	0.994	0.994	0.984	0.984	0.984
3	517	0.992	0.992	0.988	0.988	0.988
4	1433	0.991	0.992	0.988	0.987	0.987
5	668	0.997	0.997	0.990	0.990	0.990
6	472	0.994	0.994	0.992	0.989	0.989
Total	9595	0.997	0.997	0.993	0.993	0.993

**Table 5:** Root mean squared error (RMSE) between true and estimated repeat numbers with allelotype using the original BAM file of 40 × and those realigned with STR-realigner, ReviSTER, GATK IndelRealigner, and allelotype with --realign option for all the STR regions. The best result is underlined

Period	No. of regions	STR-realigner	ReviSTER	IndelRealigner	--realign option	Original BAM
1	5345	1.104	1.054	4.148	4.181	4.152
2	1160	2.638	2.679	5.454	5.762	5.477
3	517	2.778	2.746	4.818	4.768	4.818
4	1433	2.651	2.631	5.386	5.398	5.406
5	668	2.301	2.114	6.617	6.660	6.617
6	472	3.137	3.178	6.071	5.936	6.094
Total	9595	1.959	1.933	4.861	4.914	4.870

**Table 6:** Root mean squared error (RMSE) between true and estimated repeat numbers with allelotype using the original BAM file of 40 × and those realigned with STR-realigner, ReviSTER, GATK IndelRealigner, and allelotype with --realign option for commonly called STR regions. The best result is underlined

Period	No. of regions	STR-realigner	ReviSTER	IndelRealigner	--realign option	Original BAM
1	5333	1.009	0.955	4.024	4.058	4.028
2	1141	2.475	2.489	5.086	5.420	5.111
3	511	2.472	2.435	4.490	4.435	4.490
4	1414	2.270	2.371	5.157	5.152	5.177
5	661	2.058	1.865	6.263	6.308	6.263
6	467	2.476	2.977	5.888	5.747	5.912
Total	9527	1.729	1.755	4.649	4.702	4.659

In order to examine the performance on lower coverage data, we downsampled the original BAM file from 40 × to 10 × and estimated repeat numbers with RepeatSeq and allelotype using the downsampled BAM file and BAM files obtained by applying the realignment methods to the downsampled BAM file. Table 7 shows call rates of results from RepeatSeq for the downsampled BAM files. For all the STR periods other than period of 1, call rates of results from the BAM file realigned with STR-realigner are higher than those from other BAM files. Tables 8 and 9 respectively summarize RMSE values for each BAM file for all the STR regions and the regions where results were commonly estimated as a non-NA value. In the calculation of RMSE, the size in the reference genome was set for the region size estimated as NA value for Table 8. In both Tables 8 and 9, the results from the BAM file realigned with STR-realigner shows the best RMSE values for all the STR periods other than period of 1. The RMSE value from the results based on the BAM file realigned with GATK IndelRealigner is slightly better than that based on the original BAM file.

**Table 7:** Call rate of STR calling results with RepeatSeq using the original BAM file of 10 × and those realigned with STR-realigner, ReviSTER, and GATK IndelRealigner. The best result is underlined

Period	No. of regions	STR-realigner	ReviSTER	IndelRealigner	Original BAM
1	5345	0.874	0.876	0.854	0.848
2	1160	0.790	0.788	0.760	0.756
3	517	0.830	0.803	0.778	0.774
4	1433	0.831	0.759	0.735	0.736
5	668	0.859	0.793	0.774	0.774
6	472	0.871	0.824	0.807	0.814
Total	9595	0.854	0.836	0.813	0.809

**Table 8:** Root mean squared error (RMSE) between true and estimated repeat numbers with RepeatSeq using the original BAM file of 10 × and those realigned with STR-realigner, ReviSTER, and GATK IndelRealigner for all the STR regions. The best result is underlined

Period	No. of regions	STR-realigner	ReviSTER	IndelRealigner	Original BAM
1	5345	5.261	5.128	8.904	9.126
2	1160	4.779	5.984	8.879	9.162
3	517	3.441	5.409	8.629	8.880
4	1433	4.466	6.724	10.060	10.195
5	668	4.951	7.489	11.058	11.159
6	472	4.854	7.179	10.385	10.652
Total	9595	4.966	5.809	9.308	9.517

**Table 9:** Root mean squared error (RMSE) between true and estimated repeat numbers with RepeatSeq using the original BAM file of 10 × and those realigned with STR-realigner, ReviSTER, and GATK IndelRealigner for commonly called STR regions. The best result is underlined

Period	No. of regions	STR-realigner	ReviSTER	IndelRealigner	Original BAM
1	4505	5.461	5.363	9.047	9.184
2	860	4.878	6.307	9.238	9.436
3	396	3.531	4.862	8.520	8.817
4	1023	4.449	5.954	9.828	9.899
5	497	5.188	6.835	10.504	10.638
6	371	4.622	6.771	10.451	10.773
Total	7652	5.129	5.712	9.323	9.474

Tables 10, 11 and 12 show call rates, RMSE values averaged on all the regions, and RMSE values averaged on commonly called regions for results from allelotype, respectively. The results for the BAM file realigned with STR-realigner gave the highest call rate for all the STR periods other than period of 1. In total, STR-realigner gave the best results in both considering all the STR regions and commonly called regions.

**Table 10:** Call rate of STR calling results with allelotype using the original BAM file of 10 × and those realigned with STR-realigner, ReviSTER, GATK IndelRealigner, and allelotype with --realign option. The best result is underlined

Period	No. of regions	STR-realigner	ReviSTER	IndelRealigner	--realign option	Original BAM
1	5345	0.999	1.000	0.992	0.992	0.992
2	1160	0.990	0.988	0.972	0.973	0.972
3	517	0.985	0.983	0.977	0.977	0.977
4	1433	0.983	0.983	0.973	0.973	0.973
5	668	0.994	0.991	0.969	0.969	0.969
6	472	0.987	0.985	0.979	0.979	0.979
Total	9595	0.994	0.994	0.984	0.984	0.984

**Table 11:** Root mean squared error (RMSE) between true and estimated repeat numbers with allelotype using the original BAM file of 10 × and those realigned with STR-realigner, ReviSTER, GATK IndelRealigner, and allelotype with --realign option for all the STR regions. The best result is underlined

Period	No. of regions	STR-realigner	ReviSTER	IndelRealigner	--realign option	Original BAM
1	5345	2.477	2.695	6.017	6.009	6.009
2	1160	3.545	3.740	6.948	6.885	6.899
3	517	3.566	3.728	6.898	6.843	6.843
4	1433	3.754	3.818	7.476	7.417	7.431
5	668	3.910	4.469	8.760	8.762	8.762
6	472	4.319	4.265	8.232	8.217	8.233
Total	9595	3.116	3.309	6.752	6.727	6.732

**Table 12:** Root mean squared error (RMSE) between true and estimated repeat numbers with allelotype using the original BAM file of 10 × and those realigned with STR-realigner, ReviSTER, GATK IndelRealigner, and allelotype with --realign option for commonly called STR regions. The best result is underlined

Period	No. of regions	STR-realigner	ReviSTER	IndelRealigner	--realign option	Original BAM
1	5304	2.413	2.656	5.740	5.732	5.732
2	1128	3.336	3.420	6.440	6.371	6.386
3	505	3.057	3.090	6.456	6.396	6.396
4	1394	3.398	3.479	7.045	6.980	6.995
5	647	3.766	4.199	8.015	8.017	8.017
6	462	3.688	3.872	7.907	7.892	7.909
Total	9440	2.906	3.099	6.363	6.336	6.341

The results for the sequencing data of 10 × are always worse than those of 40 × in all the cases.

### Real data analysis

For real human sequencing data, we used 101 bp paired-end sequencing data of a CEU parent-offspring trio NA12878, NA12891, and NA12892 analyzed in the 1000 Genomes Project. NA12891 and NA12892 are parents of NA12878. The data was sequenced on Illumina HiSeq 2000 with the read coverage of 50 × and the average insert size of 300 bp. Sequence reads were mapped to the reference genome (GRCh37) with BWA-MEM and stored in BAM format. The data was obtained from the Illumina Platinum Genomes Project through the European Nucleotide Archive under the study accession PRJEB3381 (http://www.ebi.ac.uk/ena/data/view/ERP001960). STR-realigner, ReviSTER, and GATK IndelRealigner were applied to these BAM files. RepeatSeq was then applied to the original BAM files and these realigned BAM files and sizes of the target STR regions were estimated. In order to examine the performance, we considered the consistency in Mendelian inheritance in called regions, where the estimated region size for NA12878 is a non-NA value. STR regions used for the evaluation are the same as the regions in simulation analysis in Section 1. We counted STR regions where the estimated size for NA12878 consistent with those for her parents, NA12891 and NA12892 in terms of Mendelian inheritance as well as the STR regions with inconsistent results.

In Table 13, the number of regions with consistent sizes in terms of Mendelian inheritance (#CR) and the number of inconsistent estimation results (#IR) are summarized. Note that the larger number is better for consistent regions while the smaller number is better for inconsistent regions. The results for STR period of 1 without skipping homopolymer regions with size >15 are in parentheses.

**Table 13:** The numbers of estimated repeat numbers matched and mismatched with parents in terms of Mendelian inheritance

Period	STR-realigner		ReviSTER		IndelRealigner		Original BAM	
	#CR	#IR	#CR	#IR	#CR	#IR	#CR	#IR
1	1305	533	1319	540	1314	531	1298	531
	(1,416)	(563)						
2	280	82	269	80	242	90	242	87
3	63	5	56	7	56	6	57	5
4	196	28	183	34	169	38	169	33
5	41	15	46	18	44	16	44	15
6	35	9	34	12	33	13	33	13
Total	1920	672	1907	691	1858	694	1843	684

The number of consistent regions (#CR), and the number of inconsistent regions (#IR) based on estimated repeat numbers with RepeatSeq in a parent-offspring trio, NA12878, NA12891 and NA19892, for the original BAM files, those realigned with STR-realigner, ReviSTER, and GATK IndelRealigner are summarized. Values in parentheses for STR-realigner are the result without filtering long homopolymer regions. The best result is underlined

For STR periods of 3, 4, and 6, results from BAM files realigned with STR-realigner gave the best results in both consistent and inconsistent regions.

In total, results from BAM files realigned with STR-realigner gave the best results in both consistent and inconsistent regions. Results for GATK IndelRealigner are consistent in more regions than those for the original BAM files although the results for GATK IndelRealigner contains the most inconsistent regions.

In Table 14, the number of regions with consistent sizes in terms of Mendelian inheritance (#CR) and the number of inconsistent sizes (#IR) on results estimated with allelotype are summarized. For STR periods of 2, 3, 5, and 6, results from BAM files realigned with STR-realigner gave the best results in consistent regions. In addition, for STR periods of 2 and 5, results from BAM files realigned with STR-realigner also gave the best results in inconsistent regions. In total, the results for STR-realigner gave the best performance in both consistent and inconsistent regions.

**Table 14:** The numbers of estimated repeat numbers matched and mismatched with parents in terms of Mendelian inheritance

Period	STR-realigner		ReviSTER		IndelRealigner		--realign option		Original BAM	
	#CR	#IR	#CR	#IR	#CR	#IR	#CR	#IR	#CR	#IR
1	1772	777	1773	770	1770	773	1621	897	1775	776
	(1834)	(860)								
2	345	81	328	94	323	90	317	97	328	84
3	70	6	69	4	66	7	65	8	65	7
4	222	24	224	20	219	23	214	28	216	26
5	75	27	75	29	75	28	73	31	73	28
6	73	28	67	28	67	27	68	22	70	26
Total	2557	943	2536	945	2520	948	2358	1083	2527	947

**Fig. 4:**
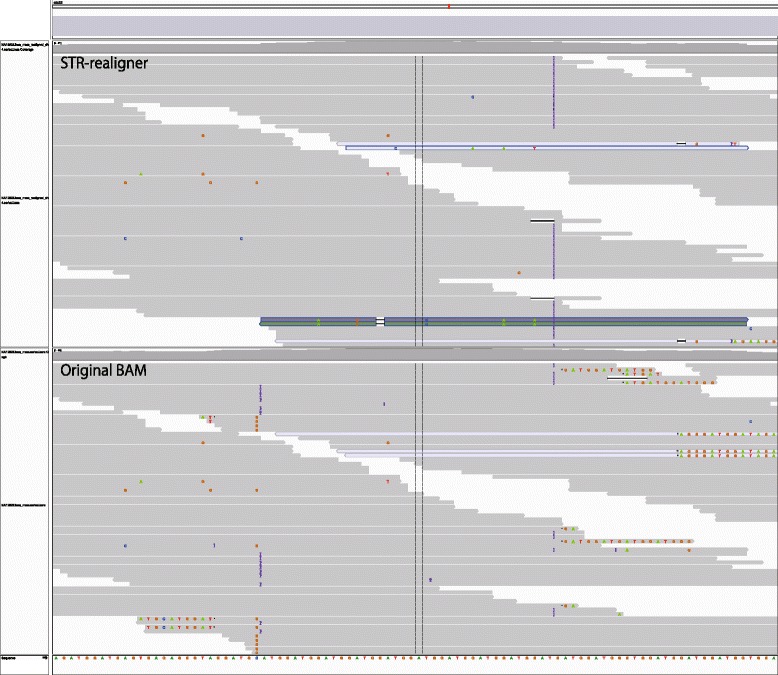
Comparison of original and realigned BAM files for NA12892 in an STR region located at chr22:28045335-28045407. The top panel is a plot of sequencing data in a BAM file realigned with STR-realigner and the bottom one is a plot of sequencing data in the original BAM file

Figure 4 shows an IGV view where STR-realigner effectively works on realigning inserted repeat patterns in an STR region comprised of GGAT repeats located at chr22:28045335-28045407 for a BAM file for NA12892. In the original BAM file, there exist clipping fragments in some reads due to the insertion of GGAT repeats, and the insertion of GGA repeats was missed with RepeatSeq, which caused the inconsistency of the estimated results in Mendelian inheritance. On the other hand, sequence reads with left clipping disappear and insertions are observed in the BAM file realigned with STR-realigner. In the estimated result with RepeatSeq, the insertion was included in the estimated size of the STR region.

We also evaluated root mean squared errors for NA12878 by using STR region sizes obtained from high coverage PacBio sequencing data as the gold standard. The PacBio sequencing data was obtained through BioProject PRJNA253696 with Sequence Read Archive accession numbers SRX627421 and SRX638310 [20] and its read coverage is 46 × in total. Error-corrected reads with Falcon (https://github.com/PacificBiosciences/FALCON) in FASTA format were aligned to GRCh37 with BWA-MEM, and STR region size data was then obtained from variant calling results by applying allelotype to the aligned reads.

Table 15 summarizes the RMSE values between estimated STR region size with RepeatSeq and the gold standard for each BAM file for all the STR regions. In the calculation of RMSE, the size in the reference genome was assigned for the region size estimated as NA value. RMSE values for estimated results with allelotype are also summarized in Table 16. In both cases using RepeatSeq and allelotype, the results from sequencing data realigned with STR-realigner showed the best performance in total.

**Table 15:** Root mean squared error (RMSE) between the gold standard and estimated STR sizes with RepeatSeq using the original BAM file for NA12878 from HiSeq 2000 and those realigned with STR-realigner, ReviSTER, and GATK IndelRealigner for all the STR regions

Period	No. of regions	STR-realigner	ReviSTER	IndelRealigner	Original BAM
1	5345	2.429	2.397	2.431	2.430
2	1160	3.587	3.860	3.829	3.804
3	517	1.901	1.902	2.000	1.998
4	1433	2.509	2.540	2.709	2.710
5	668	3.134	2.736	3.021	3.039
6	472	2.821	2.823	2.890	2.890
Total	9595	2.656	2.660	2.724	2.721

For the gold standard, STR sizes estimated from high coverage PacBio sequencing data with allelotype are used. The best result is underlined

**Table 16:** Root mean squared error (RMSE) between the gold standard and estimated STR sizes with allelotype using the original BAM file for NA12878 from HiSeq 2000 and those realigned with STR-realigner, ReviSTER, GATK IndelRealigner, and allelotype with --realign option for all the STR regions

Period	No. of regions	STR-realigner	ReviSTER	IndelRealigner	--realign option	Original BAM
1	5345	2.298	2.294	2.354	2.296	2.298
2	1160	3.152	3.293	3.265	3.243	3.129
3	517	2.033	2.039	2.034	2.038	2.034
4	1433	2.453	2.582	2.687	2.600	2.454
5	668	3.033	3.006	3.271	3.008	3.031
6	472	2.698	2.739	2.765	2.739	3.090
Total	9595	2.503	2.542	2.607	2.538	2.521

For the gold standard, STR sizes estimated from high coverage PacBio sequencing data with allelotype are used. The best result is underlined

### Comparison of computational time

Table 17 shows the computational time of STR-realigner, ReviSTER, and GATK IndelRealigner for simulation and real data analyzed in Sections 1 and 1. For the real data, the computational time for realigning the BAM file for NA12878 was measured. Computation was conducted in Intel Xeon CPU E5-2670 processors with a single thread, and computational time for each case is for single process. STR-realigner is implemented in Java. In both simulation and real data, ReviSTER required the most computational time among these methods, and STR-realigner required more computational time than GATK IndelRealigner. For STR-realigner, by limiting alignment space within some window of the original alignment result, the drastic reduction of the computational time is expected while keeping its realignment quality. In addition, the computational time can be reduced by realigning sequence reads for each STR region in a parallel manner. For memory consumption, STR-realigner requires less than 2GB in both simulation and real data.

**Table 17:** Comparison of computational time on a simulation data for an individual with read coverage of 40 × and a real dataset for NA12878

Method	Computational time	Computational time
	(Simulation data)	(Real data)
STR-realigner	2,928.90 [s]	1,186.77 [s]
ReviSTER	5,230.72 [s]	3,618.62 [s]
GATK IndelRealigner	357.46 [s]	294.13 [s]

## Conclusion

We proposed a new realignment method for STR regions named STR-realigner that takes sequence reads aligned with other methods and realigns sequence reads by dynamic programing manner with the consideration of the corresponding STR repeat pattern as prior knowledge. For the simulation data analysis, we prepared synthetically generated reads aligned with BWA-MEM, those realigned with STR-realigner, those realigned with ReviSTER, and those realigned with GATK IndelRealigner. In order to evaluate the effectiveness of our proposed method, we applied RepeatSeq and allelotype to these four types of aligned reads, and the results from sequence reads realigned with the proposed method give the best RMSE value among the results from these four types of aligned reads. From the comparison of root mean squared errors between estimated and true STR region size for these four types of aligned reads, the results for the dataset realigned with STR-realigner are better than those for other datasets for most of the cases. For real data analysis, we considered a real sequencing dataset from Illumina HiSeq 2000 for a parent-offspring trio, RepeatSeq was applied to an aligned sequencing dataset with BWA-MEM, that realigned with STR-realigner, that realigned with ReviSTER, and that realigned with GATK IndelRealigner, and the results from the dataset realigned with STR-realigner shows the best performance in terms of consistency of the size of estimated STR regions in Mendelian inheritance. In addition, by using the size for STR regions obtained from high coverage PacBio sequencing data as the gold standard, the results for STR-realigner show the best RMSE values for the case considering all the periods. In both simulation and real data, ReviSTER required the most computational time among the realignment methods considered in this work, and the proposed method required more computational time than GATK IndelRealigner. However, the computational time of STR-realigner can be reduced drastically by parallel computing and limiting the search space for the realignment around the originally aligned results in some specified window size.

## Abbreviations

BAM: Binary alignment/map
CEU: Central Europe
GATK: Genome analysis toolkit
GRC: Genome reference consortium
HTS: High-throughput sequencing
RMSE: Root mean squared error
STR: Short tandem repeat
